# Herpetic anterior uveitis following COVID-19 vaccines: a case series

**DOI:** 10.3389/fmed.2023.1242225

**Published:** 2023-09-22

**Authors:** Muriel Ott, Thanoosha Nagamany, Souska Zandi, Francesco Pichi, Aniruddha Agarwal, Ester Carreño, Vishali Gupta, Dilraj S. Grewal, Emmett T. Cunningham, Marion R. Munk

**Affiliations:** ^1^Department of Ophthalmology, Inselspital, Bern University Hospital, University of Bern, Bern, Switzerland; ^2^Cleveland Clinic Lerner College of Medicine, Case Western Reserve University, Cleveland, OH, United States; ^3^Cleveland Clinic Abu Dhabi, Eye Institute, Abu Dhabi, United Arab Emirates; ^4^Department of Ophthalmology, Maastricht University Medical Center, Maastricht, Netherlands; ^5^Department of Ophthalmology, University Hospital Fundación Jimenez Diaz, Madrid, Spain; ^6^Advanced Eye Centre, Post Graduate Institute of Medical Education and Research, Chandigarh, India; ^7^Department of Ophthalmology, Duke University, Durham, NC, United States; ^8^Department of Ophthalmology, California Pacific Medical Center, San Francisco, CA, United States; ^9^Department of Ophthalmology, School of Medicine, Stanford University, Palo Atlo, CA, United States; ^10^The Francis I. Proctor Foundation, UCSF School of Medicine, San Francisco, CA, United States; ^11^Augenarzt-Praxisgemeinschaft Gutblick AG, Pfäffikon, Switzerland; ^12^Feinberg School of Medicine, Northwestern University, Chicago, IL, United States

**Keywords:** herpetic eye disease, herpes zoster ophthalmicus, ocular herpes simplex, COVID-19 vaccines, mRNA vaccination, uveitis, keratitis, SARS-CoV-2 vaccination

## Abstract

**Purpose:**

To report a case series of herpetic uveitis following COVID-19 vaccinations.

**Methods:**

Demographic, clinical and treatment-related data of herpetic anterior uveitis cases was collected at five tertiary eye hospitals between January 2021 and June 2022. A retrospective database review at one of the centers comparing the number of cases of herpetic eye disease before and after the introduction of COVID-19 vaccination was performed as well.

**Results:**

Twenty-four patients (9 female, 15 male) with a mean age of 54 years (range 28–83 years) were diagnosed with herpetic uveitis, reporting an onset of symptoms 3–42 days after the first, second or third dose of COVID-19 vaccination. Median time between vaccination and onset of herpetic eye disease was 10 days (mean 12.7 ± 10.15 days) days. The administered vaccines were BNT162b2, mRNA-1273, BBIBP-CorV and Ad26.COV2.S. The cases included 11 HSV, 10 VZV and 1 CMV anterior uveitis, 2 were not further specified. There was an equal number of first episodes (*n* = 12, 50%) and recurrent episodes (*n* = 12, 50%). Response to established regimens was generally good. The retrospective database review revealed the exact same incidence of herpetic uveitis during the pandemic and ongoing vaccination compared to prior SARS-CoV-2.

**Conclusion:**

This report includes 24 cases of herpetic anterior uveitis in a temporal relationship to various COVID-19 vaccines. This study supports the potential risk of herpetic eye disease following COVID-19 vaccines, but proof of a direct, causal relationship is missing.

## 1. Introduction

The coronavirus disease 2019 (COVID-19) outbreak was classified as a pandemic by the World Health Organization (WHO) on 11 March 2020 (1). The effort to prevent the rapid spread of the severe acute respiratory syndrome coronavirus 2 (SARS-CoV-2), which causes COVID-19, has led to the accelerated authorization of vaccinations (2, 3). The emergence of the disease was hence followed by an unprecedented worldwide mass vaccination effort with millions of people receiving vaccinations against COVID-19. Among the administered vaccinations are the first ever approved mRNA vaccines, BNT162b2 (Pfizer-BioNTech) and mRNA−1273 (Moderna) (4, 5). Additionally, other immunizations, such as inactivated vaccines and adenovirus-based vaccines, including BBIBP-CorV (Sinopharm) and Ad26.COV2.s (Janssen), have been developed and used (6, 7).

The benefits of a vaccination always come with the potential of a wide range of side effects. The temporal association between uveitis and vaccinations is well established in the literature (8). Numerous cases of vaccine-associated uveitis are described after globally administered vaccines, such as influenza, hepatitis B, human papilloma virus (HPV) and measles-mumps-rubella (MMR) vaccines (8–13). In that sense, during the last 2 years, reports of uveitis cases after COVID-19 vaccinations have accumulated. The majority of the cases were mild and showed a good treatment response, while more complicated courses with posterior involvement were only rarely described (14–16).

The reactivation of herpes viruses seems to be another plausible risk of COVID-19 vaccines: Several cases of COVID-19 vaccine associated varicella zoster virus (VZV) and herpes simplex virus (HSV) reactivations have been reported (17–21). Additionally, analyses of pharmacovigilance databases indicate a risk of herpesvirus reactivations after COVID-19 mRNA vaccines (22, 23).

This article presents 24 cases of herpetic anterior uveitis following COVID-19 vaccination. We have also assessed the mean frequency of herpetic eye diseases prior to the SARS-CoV-2 pandemic and following the introduction of vaccination.

## 2. Methods

### 2.1. Study design

This study was a multicenter, retrospective case series. The study complied with the Declaration of Helsinki and was approved by the Ethics committee (Basec ID: 2019–01590). Institutional Review Board approval was also determined by the respective coauthors according to the requirements established by their institutional centers.

### 2.2. Patients and clinical samples

Patients with a diagnosis of herpetic eye disease presenting 1 day to 5 weeks after a COVID-19 vaccination at one of the participating five tertiary eye care centers were included. Diagnosis was either microbiologically confirmed or was clinically so characteristic that in the discretion of the treating uveitis specialist no microbiological confirmation was necessary. Cases without uveitis (e.g., skin lesions only) were excluded. All included cases were seen between January 2021 and June 2022.

Demographics, clinical and treatment-related characteristics were collected based on medical records.

Vaccinations were documented as azd1222 (AstraZeneca), BNT162b2 (Pfizer-BioNTech), mRNA−1,273 (Moderna), Ad26.COV2.S (Janssen COVID-19 Vaccine) or other, additionally note was taken of the delay in days and after which dose the herpetic eye disease occurred.

It was indicated if the patient had suffered previous reactivations or if it was the first episode. Furthermore, the presence or absence of various forms of periocular skin lesions, keratitis and uveitis was reported; the latter being classified according to the Standardization of Uveitis Nomenclature (SUN) criteria. Anterior HSV uveitis, anterior VZV uveitis, acute retinal necrosis (ARN), progressive outer retinal necrosis (PORN), anterior CMV uveitis, CMV retinitis and herpetic anterior uveitis (species unknown) were differentiated.

In addition, the intraocular pressure (IOP) and the best corrected visual acuity (BCVA, expressed in decimals) at the initial presentation were obtained.

The data collection included possible relevant pre-existing co-morbidities, prophylactic antiviral and immunosuppressive therapies as well as the initiated treatment at the time of diagnosis and a description of the follow-up ophthalmological history.

The likelihood of whether the herpetic anterior uveitis happened due to the administered COVID-19 vaccination or rather resulted because of other factors was evaluated using the Naranjo Scale (24).

We additionally compared the incidence of herpetic eye diseases in one clinic (University Hospital of Bern) prior SARS-CoV-2 onset and during the pandemic. This assessment was based on patient lists generated by the search of all electronic medical records for the German keywords “herpetisch” (in English: herpetic), and the ICD10 codes “Herpes simplex, Auge” (in English: Herpes Simplex, Eye) and “Herpes Zoster ophthalmicus, mit Augenbeteiligung” (in English: Herpes Zoster ophthalmicus, with ocular involvement).

### 2.3. Statistical analysis

Statistical analysis was performed using IBM^®^ SPSS^®^ Statistics (Version: 28.0.1.1 [14]). Continuous variables were reported as mean, standard deviation (SD), and median and range when appropriate. Categorical variables were reported as percentages. Frequency of herpetic eye diseases prior and during the pandemic were compared using Binominal testing. For this test, statistical significance was indicated by *p* ≤ 0.05.

## 3. Results

### 3.1. Patient characteristics

A total of 29 patients with herpetic eye disease were registered. Five subjects presented with either skin lesions (*n* = 1) or keratitis (*n* = 4) only and were excluded. Of the remaining 24 patients, 15 (62.5%) were males and 9 (37.5%) females, with a mean age of 53.9 ± 16 years (range: 28–83, median: 50).

Six patients (25%) were each vaccinated with BNT162b2 and Ad26.COV2.S, respectively, 3 (12.5%) with mRNA−1273. Among the 9 patients (37.5%) reported to have received yet a different vaccination, 6 were vaccinated with BBIBP-CorV. The residual 3 received either BNT162B2 or mRNA−1237, however which one exactly was not remembered and remained unclear. None of the registered patients received azd1222.

Six (25%) of the herpes reactivations were diagnosed after the 1st, 11 (45.8%) after the 2nd and 7 (29.2%) after the 3rd dose. None of the reported cases happened after a 4th dose.

Patients reported first symptoms 3–42 days after the vaccination with a median delay of 10 days (mean 12.7 ± 10.15 days).

There was an equal number of first episodes (*n* = 12, 50%) and recurrent episodes (*n* = 12, 50%).

Eleven (45.8%) of the cases were classified as anterior HSV and 10 (41.7%) as anterior VZV uveitis, 2 cases (8.3%) were described as uveitis with high suspicion of herpetic origin without making a more specific diagnosis. One case had an anterior CMV uveitis, who was also the only patient under prophylactic antiviral therapy.

No cases of necrotizing herpetic retinitis or herpetic chorioretinitis were observed during the period of the study.

Most of the cases (*n* = 20, 83.3%) were diagnosed based on a characteristic clinical picture. In one case (4.2%) the clinical presentation was rather atypical, but the patient showed a good response to antiviral treatment. In 3 cases (12.5%) the diagnosis was confirmed by polymerase chain reaction (PCR) analysis of aqueous humor; among them a first episode of an anterior VZV uveitis (without skin lesions), a recurrence of an anterior HSV uveitis and the only reported CMV case.

Relevant or probably relevant co-morbidities (without further specification) were indicated for 3 cases (12.5%), among them the patient with the anterior CMV uveitis.

No patient was under treatment with a disease modifying antirheumatic drug at the time of diagnosis of the reported herpetic eye disease.

### 3.2. Clinical presentation

Most patients presented with moderate anterior chamber cells (1+: *n* = 8 [33.3%], 2+: *n* = 10 [41.7%], 5 patients [20.8%] had 3+ and 1 patient [4.2%] had 0.5+ cells). Anterior chamber flare was indicated as absent in 11 cases (45.8%), as 1+ in another 11 cases (45.85%) and as 2+ in 2 cases (8.3%).

The worst reported BCVA was hand-motion (*n* = 1, 4.2%). The residual 23 patients (95.8%) had a mean BCVA of 0.57 ± 0.44 (minimum: 0.05, maximum: 1.25, median: 0.5).

IOP elevation (>21 mmHg) as a common feature of anterior herpetic uveitis was observed in 13 cases (54.17%), the mean IOP was 24 ± 12 mmHg (range: 8–60, median: 22.5). Granulomatous keratic precipitates were described in 15 cases (62.5%).

Four (16.7%) of the uveitis cases presented with a concurrent keratitis. Typical herpetic skin lesions were indicated in 4 patients (16.7%), 3 of them had anterior VZV uveitis.

### 3.3. Treatment and follow-up

The follow-up period ranged between 1 and 36 weeks (mean: 14.59 ± 10.87, median: 14). Information on follow-up periods was not provided for 7 cases (equals all the cases from Eye Institute, Cleveland Clinic Abu Dhabi), with the remark that the follow-up was still ongoing. Three patients (all from University Hospital of Bern) were lost to follow-up.

Twenty-three patients (95.5%) received topical steroids and oral antiviral therapy. One patient with +0.5 anterior chamber cells received only antiviral therapy.

Information on treatment response was forwarded for 16 cases: Twelve (75%) showed a good response with fast tapering, 3 (18.8%) showed a mediocre response with slow tapering and 1 (6.3%) showed a poor response and required intensification of treatment.

Twenty-three patients (95.5%) received oral valacyclovir. The anterior CMV uveitis case was treated with topical ganciclovir. The above-mentioned patient with poor treatment response developed a herpetic cerebral vasculitis and was switched from oral valacyclovir to intravenous acyclovir.

Information on prophylaxis was missing for the 7 cases without information on the duration of the follow-up period (equals all the cases of from Eye Institute, Cleveland Clinic Abu Dhabi). Seven (41.2%) of the residual 17 cases, were started on long-term (minimal duration not further specified) antiviral prophylaxis, among them 4 first episodes (2 anterior VZV uveitis, 2 not further specified anterior herpetic uveitis) and 3 recurrent episodes (2 anterior HSV uveitis, 1 anterior VZV uveitis).

### 3.4. Causality assessment

The Naranjo Scale for each case was two, indicating a possible causal relationship (24). Details are shown in Tables 1, 2.

**Table 1:** Naranjo Scale—items and scores (24).

Question	Score		
	Yes	No	Do not know
1. Are there previous conclusive reports on this reaction?	+1	0	0
2. Did the adverse event appear after the suspected drug was administered?	+2	−1	0
3. Did the adverse event improve when the drug was discontinued or a specific antagonist was administered?	+1	0	0
4. Did the adverse event reappear when the drug was readministered?	+2	−1	0
5. Are there alternative causes that could on their own have caused the reaction?	−1	+2	0
6. Did the reaction reappear when a placebo was given?	−1	+1	0
7. Was the drug detected in blood or other fluids in concentrations known to be toxic?	+1	0	0
8. Was the reaction more severe when the dose was increased or less severe when the dose was decreased?	+1	0	0
9. Did the patient have a similar reaction to the same or similar drugs in any previous exposure?	+1	0	0
10. Was the adverse event confirmed by any objective evidence?	+1	0	0

**Table 2:** Score calculation and total score for each case following the Naranjo Scale.

Case	Q1	Q2	Q3	Q4	Q5	Q6	Q7	Q8	Q9	Q10	Total score
1	0	2	0	0	−1	0	0	0	0	1	2
2	0	2	0	0	−1	0	0	0	0	1	2
3	0	2	0	0	−1	0	0	0	0	1	2
4	0	2	0	0	−1	0	0	0	0	1	2
5	0	2	0	0	−1	0	0	0	0	1	2
6	0	2	0	0	−1	0	0	0	0	1	2
7	0	2	0	0	−1	0	0	0	0	1	2
8	0	2	0	0	−1	0	0	0	0	1	2
9	0	2	0	0	−1	0	0	0	0	1	2
10	0	2	0	0	−1	0	0	0	0	1	2
11	0	2	0	0	−1	0	0	0	0	1	2
12	0	2	0	0	−1	0	0	0	0	1	2
13	0	2	0	0	−1	0	0	0	0	1	2
14	0	2	0	0	−1	0	0	0	0	1	2
15	0	2	0	0	−1	0	0	0	0	1	2
16	0	2	0	0	−1	0	0	0	0	1	2
17	0	2	0	0	−1	0	0	0	0	1	2
18	0	2	0	0	−1	0	0	0	0	1	2
19	0	2	0	0	−1	0	0	0	0	1	2
20	0	2	0	0	−1	0	0	0	0	1	2
21	0	2	0	0	−1	0	0	0	0	1	2
22	0	2	0	0	−1	0	0	0	0	1	2
23	0	2	0	0	−1	0	0	0	0	1	2
24	0	2	0	0	−1	0	0	0	0	1	2

Interpretation of total scores: 0 = doubtful adverse drug reaction (DR), 1–4 = possible ADR, 5–8 = probable ADR, ≥9 = definite ADR (24).

### 3.5. Incidence of herpetic eye diseases prior and during SARS-CoV-2

We compared the incidence of herpetic uveitis diseases at the University Hospital of Bern between 01/2019–12/2019 prior the pandemic and 01/2021–12/2021 during the pandemic when vaccination was well underway in Switzerland, respectively. Eighty cases of herpetic uveitic eye diseases were reported at the University Hospital of Bern from January–December 2019. The exact same number was seen between 01/2021–12/2021 (binominal test *p* = 1.0).

## 4. Discussion

### 4.1. Main results

We presented 24 cases of herpetic uveitis following COVID-19 vaccination. Fifty percent of the patients were immunized with one of the mRNA vaccines (BNT162b2 and mRNA−1273), while the other patients received either a vector-based (Ad26.COV2.S) or an inactivated (BBIBP-CorV) vaccine. Most cases were classified as either HSV or VZV anterior uveitis. Only one case of CMV anterior uveitis was reported, no case of posterior uveitis was registered. The majority of the cases presented with one or several typical features of herpetic uveitis such as IOP elevation, KPs, concomitant corneal and skin involvement. Overall, response to treatment with oral valacyclovir and topical steroids was good. One patient needed intravenous valacyclovir due to the development of a cerebral herpes zoster vasculitis. The only reported case of CMV anterior uveitis was successfully treated with topical ganciclovir and topical steroids.

The retrospective database review of one of the participating centers exhibited the exact same incidence of herpetic uveitis within a one-year period prior the pandemic compared to a one-year period during the pandemic and ongoing vaccination.

### 4.2. Findings in relation to other studies

In 2021, a study from the largest health care organization in Israel analyzed data on individuals vaccinated with the mRNA COVID-19 vaccine BNT162b2 and compared to unvaccinated ones. The vaccinated individuals showed a substantially higher risk for herpes zoster infections (23). This is in line with an association study between herpes zoster reporting and mRNA COVID-19 vaccines (BNT162b2 and mRNA-1273) using an international pharmacovigilance database. This study concluded that herpes zoster was more frequently reported after the mRNA COVID-19 vaccines than after influenza vaccines. Yet, considering the amount of administered mRNA vaccine doses, the number of herpes zoster cases were still low (22). Apart from herpetic disease in the context of COVID-19 vaccinations, there are also case reports on herpes simplex and zoster reactivations after other vaccines (hepatitis A, influenza, and rabies) (25–27).

Three other registry-based studies reported not only herpes zoster but multiple herpes simplex cases after COVID-19 shots (BNT162b2, mRNA-1273, and AZD1222) (18, 28, 29). Our study confirms an equal number of VZV as well as HSV reactivations after mRNA vaccines. We also documented several cases of VZV uveitis after the administration of an inactivated vaccine (BBIBP-CorV), which supports a possible risk of reactivation for both mentioned herpes viruses after COVID-19 vaccines.

Apart from VZV and HSV reactivations we also had a case of anterior CMV uveitis in our study. So far there is only one other published case of an ocular CMV reactivation after COVID-19 immunization with a vaccine (15), but several extraocular CMV infections after COVID-vaccines were reported (30–32).

The mean age in our study was 54 years. This is comparable to the mean age of herpetic anterior uveitis in other studies, where uveitis was not related to COVID-19 vaccination (33, 34). Also, in terms of treatment response there was no relevant difference to vaccine-unrelated herpetic anterior uveitis cases; the hereby reported vaccine-related HSV and VZV anterior uveitis responded well to the established herpetic uveitis treatment with topical steroids and oral valacyclovir (35, 36).

It is known, that the history of herpes zoster and simplex infection is vastly influenced by the immune status of the host (17). In our case series only 5 patients were described as suffering from a potentially relevant co-morbidity and none of the patients was under systemic immunosuppression. Hence, there is no obvious difference in the risk of herpetic uveitis after COVID-19 vaccines in healthy individuals compared to the risk in immunocompromised patients. It suggests that the vaccination may just act as an additional trigger for activation.

Data from dermatological herpes manifestations suggest a higher risk of reactivation after the first dose compared to the second dose of mRNA vaccines. Most of the cases happened during the first 2 weeks after the shots (18, 22, 37). Our case series had more cases after the second, than after the first or third dose. However, no significant conclusions can be drawn here, due to the heterogeneity of applied vaccines and the small number of included cases. Also, we initiated the study when most of the people received their second or third shots with probably already heightened awareness of such potential side effects and patients were proactively asked about potential vaccinations. The median delay of 10 days after the application of the vaccines in our study is in accordance with previous published herpes reactivation after COVID-19 vaccines (22). However, a single case of our study presented with symptoms 42 days after the vaccination. Such a long time period between the vaccination and the onset of symptoms, makes a causal relationship unlikely.

Nonetheless, the impact of the vaccination may not be very strong. The retrospective database review of one of the participating centers exhibited no higher incidence of herpetic eye diseases pre compared to post SARS-CoV-2.

### 4.3. Implications for future research and clinicians

Each of the COVID-19 vaccines reported in this study underwent large phase III clinical trials and confirmed their efficacy and satisfactory safety profiles. In none of the phase III trials was any case of uveitis registered—including cases of herpetic anterior uveitis. The participants were specifically asked about certain potential side effects during the first 7 and up to 28 days after each dose. These solicited adverse events did not include eye complaints. Beside the registration of the solicited events, only health problems that led to a medical consultation were documented. This raises the question of a possible lack of reporting or a very low incidence of ocular events (6, 7, 38, 39). Limited follow-up duration and sample size as well as enrolment restrictions in even large clinical trials lead to inherent constraints (40). Therefore, post marketing surveillance is essential to identify less common adverse events and identify certain patient groups of increased risk.

Various studies used published literature and adverse event reporting databases to assess systematically the potential of ocular adverse events since the introduction of COVID-19 vaccines (14, 41–43). In addition to the documentation of herpes zoster ophthalmicus and ocular herpes simplex, cases of autoimmune mediated, mainly mild anterior uveitis after BNT162b2, mRNA-1273, AZD1222, and Ad26.COV.S have been reported and are of special interest to this study (41, 43–45).

The potential association between vaccines and uveitis is not a new finding. Vaccine-associated uveitis (VAU) has been reported after the administration of several established vaccines, including diphtheria-pertussis-tetanus (dpt), bacilli Calmette-Guerin (BCG), hepatitis A, hepatitis B, human papillomavirus (HPV), influenza, measles, measles-mumps-rubella (MMR), pneumococcal and pox viral particles vaccines. VAU were predominately described to be anterior, mild and with a good response to treatment. Unfortunately, larger reports on VAU often omitted details on treatment regimens and on features like IOP, possible KPs as well as corneal and skin lesions that might allow conclusions on the probability of a purely autoimmune versus a herpetic uveitis (8, 46). The diagnosis of herpetic anterior uveitis in our study in distinction to purely autoimmune mediated VAU was mainly based on the typical clinical presentation with IOP elevation, KPs, keratitis or skin lesions, supported by an overall good response to antiviral treatments.

Considering a causal relationship, the exact mechanisms of herpetic disease and herpetic anterior uveitis after vaccinations remain unclear. Vaccine-induced immunomodulation may play a role. An adequate T-cell response is essential to control latent viral infections like VZV, HSV, and CMV. A shift in the T-cell population toward CD8+ and CD4+ T-cells specific for antigens of the vaccination could lead to a temporarily weakening of the herpes specific T-cells, hence posing the risk of reactivation (47). Vaccination may also induce an imbalance of CD4+ T cells and natural killer cells, as well as a dysfunction in cytokine signaling. Another potential explanation may be the mild stress of the acute phase reaction induced by the vaccination.

### 4.4. Strengths and weaknesses

To the best of our knowledge, this is the largest case series of herpetic anterior uveitis following COVID-19 vaccines published so far. Thus, in a broader sense this study contributes to a better understanding of herpetic eye diseases and helps to raise awareness of a possible increased risk of herpes reactivations after COVID-19 vaccinations.

The lack of laboratory confirmation (anterior chamber tap, PCR) in many cases comes with an uncertainty of diagnosis, especially when differentiating VZV and HSV uveitis in patients without skin rashes.

Although general information on the course of disease and treatment was collected, detailed data on the treatment response, including the final visual acuity, is missing.

Since this study is a retrospective case series, no estimation of the risk of herpetic uveitis after COVID-19 vaccines is possible. No information on re-administration of vaccines and potential re-activation of herpetic AU is available (Naranjo Score 0 in question 4). This information of course would significantly help to understand the causality between vaccination and herpetic AU.

Only a single center assessed the incidence of herpetic AU, which is of course error prone. Assessments using large dataset like the IRIS registry may be useful, to confirm our observation. Last but not least, we compared the incidence of herpetic AU 2019 to the incidence 2021. The main outbreak in Switzerland started March 2020. Nonetheless it is likely that some infections occurred already end of 2019, which may confound our assessment.

### 4.5. Conclusion

Our study demonstrated a variable temporal relationship between COVID-19 vaccination and herpetic anterior uveitis in a small cohort of individuals. No association between the COVID-19 vaccine and herpetic anterior uveitis was demonstrated at population level, furthermore studies to demonstrate a definitive causal relationship are missing. The cumulative strength of evidence for a causal relationship in each case using the well-accepted Naranjo Scale was uniformly categorized as “possible.” Moreover, a comparison of the number of cases of herpetic anterior uveitis seen at one of the contributing clinics prior to and following the introduction of COVID-19 immunization revealed no difference in incidence, weakening, as opposed to strengthening, the case in support of such a causal association. We encourage to differentiate between autoimmune vaccine-associated uveitis and herpetic uveitis, since treatment strategies are different. We advise clinicians to be aware of the possible risk of herpesvirus reactivations after COVID-19 vaccinations, including ocular manifestations such as keratitis and uveitis, and to start appropriate treatment if a reactivation is suspected. It must be noted though, that a history of herpetic eye disease is not a contraindication to COVID-19 vaccines and that the reported herpetic anterior uveitis cases responded well to standard treatment. Additionally, there is no evidence for prophylactic antiviral therapy for patients with a history of herpetic eye diseases undergoing COVID-19 immunization. In conclusion, we recommend to encourage patients who developed a herpetic anterior uveitis after a COVID-19 vaccination to complete their course of immunization. Nonetheless provided a thorough and individual risk–benefit evaluation, prophylactic antiviral treatment could be considered for patients with multiple or complex previous episodes of herpetic eye diseases.

## Data availability statement

The raw data supporting the conclusions of this article will be made available by the authors, without undue reservation if requested.

## Ethics statement

The studies involving humans were approved by the Ethics Committee (Basec ID: 2019–01590). Institutional Review Board approval was also determined by the respective coauthors according to the requirements established by their institutional centers. The studies were conducted in accordance with the local legislation and institutional requirements. Written informed consent for participation was not required from the participants or the participants’ legal guardians/next of kin because this study is a retrospective case series without involvement of personally identifiable information.

## Author contributions

MO initiated the study together with MM, and was responsible for data collection, analysis and drafting of the manuscript. SZ, TN, FP, EC, DG, VG, and AA participated by contributing cases and critically reviewing the manuscript. ETC critically reviewed the manuscript. MM supervised the study and gave significant intellectual input to the project. All authors contributed to the article and approved the submitted version.
